# 2-Amino-4,6-di­methyl­pyrimidine–sorbic acid (1/1)

**DOI:** 10.1107/S1600536813018175

**Published:** 2013-07-10

**Authors:** Sundaramoorthy Gomathi, Packianathan Thomas Muthiah

**Affiliations:** a School of Chemistry, Bharathidasan University, Tiruchirappalli 620 024, Tamilnadu, India

## Abstract

In the crystal of the title compound, C_6_H_9_N_3_·C_6_H_8_O_2_, the 2-amino-4,6-di­methyl­pyrimidine and sorbic acid mol­ecules are linked through N—H⋯O and O—H⋯N hydrogen bonds, which generate a cyclic bimolecular heterosynthon with an *R*_2_^2^(8) graph-set motif. Further, two inversion-related pyrimidine mol­ecules are base-paired *via* a pair of N—H⋯N hydrogen bonds, forming a cyclic bimolecular homosynthon with a graph-set of *R*_2_^2^(8). A discrete hetero tetra­meric supra­molecular unit along the *b* axis is formed by the fusion of two heterosynthons and one homosynthon. An aromatic π–π inter­action [centroid–centroid distance = 3.7945 (16) Å] is observed between these tetra­meric units.

## Related literature

For amino­pyrimidine–carb­oxy­lic acid inter­actions, see: Hunt *et al.* (1980 ▶). For related structures, see: Thanigaimani *et al.* (2007 ▶); Ebenezer & Mu­thiah (2010 ▶, 2012 ▶). For hydrogen-bond motifs, see: Bernstein *et al.* (1995 ▶); Etter (1990 ▶).
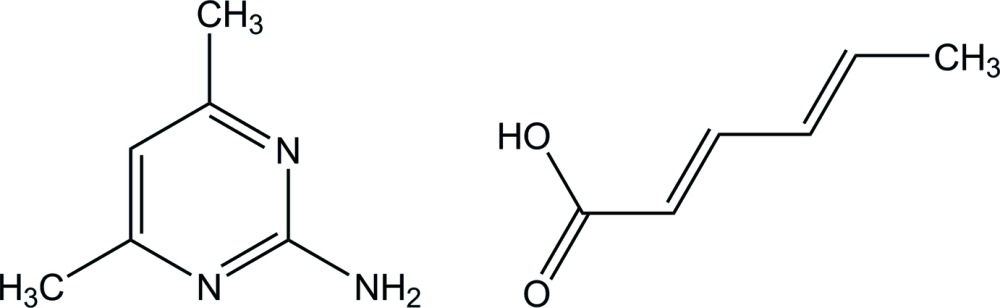


## Experimental

### 

#### Crystal data

C_6_H_9_N_3_·C_6_H_8_O_2_
*M* *_r_* = 235.29
Triclinic, 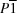

*a* = 7.8441 (6) Å
*b* = 9.9413 (8) Å
*c* = 10.2846 (13) Å
α = 112.058 (7)°
β = 98.333 (8)°
γ = 111.306 (5)°
*V* = 654.69 (13) Å^3^
*Z* = 2
Mo *K*α radiation
μ = 0.08 mm^−1^
*T* = 296 K
0.12 × 0.11 × 0.09 mm

#### Data collection

Bruker SMART APEXII CCD area-detector diffractometer
Absorption correction: multi-scan (*SADABS*; Bruker, 2008 ▶) *T*_min_ = 0.990, *T*_max_ = 0.993
9667 measured reflections
2280 independent reflections
1585 reflections with *I* > 2σ(*I*)
*R*_int_ = 0.048

#### Refinement

*R*[*F* ^2^ > 2σ(*F* ^2^)] = 0.069
*wR*(*F* ^2^) = 0.210
*S* = 1.03
2280 reflections
169 parameters
H atoms treated by a mixture of independent and constrained refinement
Δρ_max_ = 0.27 e Å^−3^
Δρ_min_ = −0.28 e Å^−3^

### 

Data collection: *APEX2* (Bruker, 2008 ▶); cell refinement: *SAINT* (Bruker, 2008 ▶); data reduction: *SAINT*; program(s) used to solve structure: *SHELXS97* (Sheldrick, 2008 ▶); program(s) used to refine structure: *SHELXL97* (Sheldrick, 2008 ▶); molecular graphics: *PLATON* (Spek, 2009 ▶), *Mercury* (Macrae *et al.*, 2008 ▶) and *POV-RAY* (Cason, 2004 ▶); software used to prepare material for publication: *PLATON*.

## Supplementary Material

Crystal structure: contains datablock(s) global, I. DOI: 10.1107/S1600536813018175/is5286sup1.cif

Structure factors: contains datablock(s) I. DOI: 10.1107/S1600536813018175/is5286Isup2.hkl

Additional supplementary materials:  crystallographic information; 3D view; checkCIF report

## Figures and Tables

**Table 1:** Hydrogen-bond geometry (Å, °)

*D*—H⋯*A*	*D*—H	H⋯*A*	*D*⋯*A*	*D*—H⋯*A*
O2—H2⋯N1	0.99 (4)	1.70 (4)	2.674 (3)	167 (4)
N2—H2*A*⋯N3^i^	0.89 (3)	2.19 (3)	3.076 (4)	176 (2)
N2—H2*B*⋯O1	0.86 (4)	2.10 (4)	2.946 (4)	171 (3)

Symmetry code: (i) 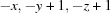.

**Fig. 1.**
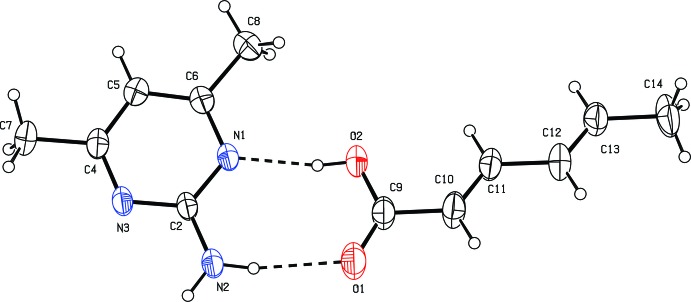
The asymmetric unit of the title compound, shown in 30% probability displacement ellipsoids.

**Fig. 2.**
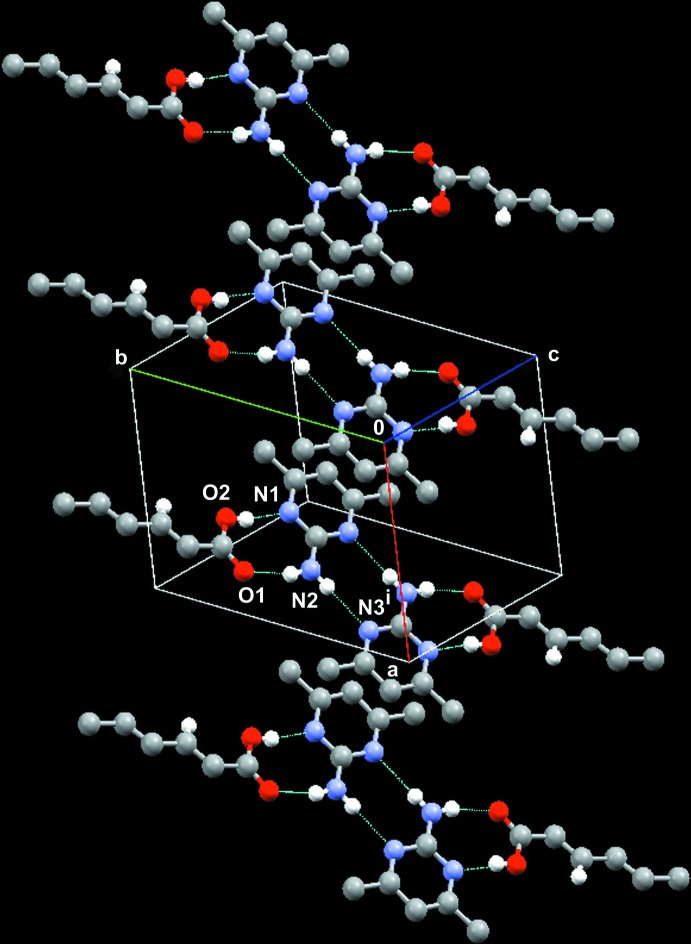
A view of supramolecular sheets formed by linear hetero tetramer. [Symmetry code: (i) -x, 1 - y, 1 - z.]

**Fig. 3.**
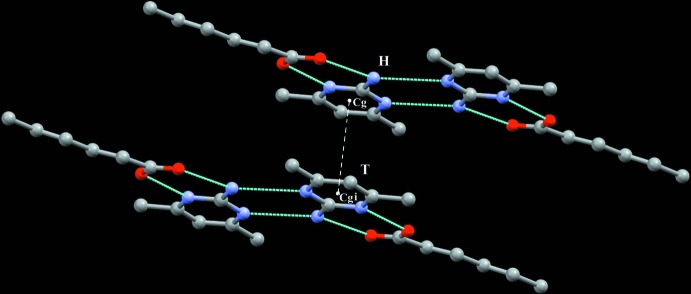
A view of aromatic π–π stacking interaction between two parallel planes. [Symmetry code: (i) 1 - x, 1 - y, 1 - z.]

## supplementary crystallographic information

### Comment

The non-covalent interactions of aminopyrimidine with carboxylic acid derivatives are of immense significance, since they involve in many molecular recognition process of biological functions and protein-drug binding (Hunt *et al.*,1980). Sorbic acid is an antibacterial agent and widely used as a preservatives. Several salts and co-crystals involving 2-amino-4,6-dimethoxy/dimethyl pyrimidine and various carboxylates (Ebenezer & Muthiah, 2010) have already been reported from our laboratory.

The current investigation focuses on the supramolecular hydrogen-bonded patterns exhibited by the (1:1) co-crystal of 2-amino-4,6-dimethylpyrimidine with sorbic acid. The asymmetric unit of the titled co-crystal consists of one molecule of 2-amino-4,6-dimethylpyrimidine (AMPY) and a molecule of sorbic acid (SA) (Fig. 1). The SA molecule exists in the EE configuration. The extended conformation of SA can be inferred from the four torsion angles, C9—C10—C11—C12 = -178.1 (3)°, C10—C11—C12—C13 = 175.5 (3)°, C11—C12—C13—C14 = -179.0 (3)° and O1—C9—C10—C11 = 168.8 (3)°. The values are in close agreement with those in the literature (Thanigaimani *et al.*, 2007).

The primary supramolecular synthon is assembled *via* N—H···O and O—H···N hydrogen bonds between the carboxylic group of SA and the amino pyrimidine moiety of AMPY to form a cyclic bimolecular heterosynthon with an *R*_2_^2^(8) graph-set motif (Etter, 1990; Bernstein *et al.*, 1995). Two centrosymmetric AMPY molecules are self-assembled to form complementary base pairing *via* a pair of N—H···N hydrogen bonds to form another *R*_2_^2^(8) ring motif. The complementary base pairing involves 2-amino group and ring N3^i^ atom of inversion related pyrimidine moiety of AMPY. The primary and secondary interactions lead to the generation of a discrete and stable linear hetero tetramer along the *b* axis (Ebenezer & Muthiah, 2012) which is a four-component supramolecule formed by the fusion of two centrosymmetric bimolecular heterosynthons [*R*_2_^2^(8)] and a homosynthon [*R*_2_^2^(8)] (Fig. 2). These discrete linear hetero tetrameric units are arranged in two dimensional space as sheets without any neighbouring interactions in the same plane.

The pyrimidine moiety of inversion related linear heterotetrameric units present in the parallel planes are stacked by an aromatic π–π interaction in a head to tail fashion (Fig. 3) with the interplanar distance of 3.580 Å, centroid to centroid distance of 3.7945 (16) Å [*Cg*–*Cg*^i^; symmetry code: (i) 1 - *x*,1 - *y*,1 - *z*] and the slip angle of 19.36°.

### Experimental

Hot aqueous solutions of 2-amino-4, 6-dimethylpyrimidine (31 mg, Aldrich) and sorbic acid (28 mg, Sisco) were mixed in a 1:1 molar ratio. The resulting solution was warmed over a water bath for half an hour and then kept at room temperature for crystallization. After a week, colorless prismatic crystals were obtained.

### Refinement

The hydrogen atoms for NH_2_ and OH groups were located in a difference Fourier map and refined freely. All other hydrogen atoms were positioned geometrically (C—H = 0.93–0.96 Å) and were refined using a riding model, with *U*_iso_(H) = 1.2*U*_eq_(C) for CH or 1.5*U*_eq_(C) for CH_3_.

### Crystal data

**Table:**

C_6_H_9_N_3_·C_6_H_8_O_2_	*Z* = 2
*M**_r_* = 235.29	*F*(000) = 252
Triclinic, *P*1	*D*_x_ = 1.194 Mg m^−^^3^
Hall symbol: -P 1	Mo *K*α radiation, λ = 0.71073 Å
*a* = 7.8441 (6) Å	Cell parameters from 2280 reflections
*b* = 9.9413 (8) Å	θ = 2.3–25.1°
*c* = 10.2846 (13) Å	µ = 0.08 mm^−^^1^
α = 112.058 (7)°	*T* = 296 K
β = 98.333 (8)°	Prism, colourless
γ = 111.306 (5)°	0.12 × 0.11 × 0.09 mm
*V* = 654.69 (13) Å^3^	

### Data collection

**Table:**

Bruker SMART APEXII CCD area-detector diffractometer	2280 independent reflections
Radiation source: fine-focus sealed tube	1585 reflections with *I* > 2σ(*I*)
Graphite monochromator	*R*_int_ = 0.048
φ and ω scans	θ_max_ = 25.1°, θ_min_ = 2.3°
Absorption correction: multi-scan (*SADABS*; Bruker, 2008)	*h* = −9→9
*T*_min_ = 0.990, *T*_max_ = 0.993	*k* = −11→11
9667 measured reflections	*l* = −12→12

### Refinement

**Table:**

Refinement on *F*^2^	Primary atom site location: structure-invariant direct methods
Least-squares matrix: full	Secondary atom site location: difference Fourier map
*R*[*F*^2^ > 2σ(*F*^2^)] = 0.069	Hydrogen site location: inferred from neighbouring sites
*wR*(*F*^2^) = 0.210	H atoms treated by a mixture of independent and constrained refinement
*S* = 1.03	*w* = 1/[σ^2^(*F*_o_^2^) + (0.1185*P*)^2^ + 0.152*P*] where *P* = (*F*_o_^2^ + 2*F*_c_^2^)/3
2280 reflections	(Δ/σ)_max_ < 0.001
169 parameters	Δρ_max_ = 0.27 e Å^−^^3^
0 restraints	Δρ_min_ = −0.28 e Å^−^^3^

### Special details

**Table:**

Geometry. Bond distances, angles *etc*. have been calculated using the rounded fractional coordinates. All su's are estimated from the variances of the (full) variance-covariance matrix. The cell e.s.d.'s are taken into account in the estimation of distances, angles and torsion angles
Refinement. Refinement on *F*^2^ for ALL reflections except those flagged by the user for potential systematic errors. Weighted *R*-factors *wR* and all goodnesses of fit *S* are based on *F*^2^, conventional *R*-factors *R* are based on *F*, with *F* set to zero for negative *F*^2^. The observed criterion of *F*^2^ > σ(*F*^2^) is used only for calculating -*R*-factor-obs *etc*. and is not relevant to the choice of reflections for refinement. *R*-factors based on *F*^2^ are statistically about twice as large as those based on *F*, and *R*-factors based on ALL data will be even larger.

### Fractional atomic coordinates and isotropic or equivalent isotropic displacement parameters (Å^2^)

**Table:**

	*x*	*y*	*z*	*U*_iso_*/*U*_eq_	
N1	0.2301 (3)	0.2463 (2)	0.49425 (19)	0.0543 (7)	
N2	0.0413 (4)	0.3650 (3)	0.5821 (3)	0.0802 (10)	
N3	0.1624 (3)	0.4209 (2)	0.40922 (19)	0.0554 (7)	
C2	0.1464 (3)	0.3438 (3)	0.4937 (2)	0.0539 (7)	
C4	0.2658 (3)	0.3961 (3)	0.3191 (2)	0.0567 (8)	
C5	0.3524 (4)	0.2964 (3)	0.3122 (3)	0.0652 (9)	
C6	0.3326 (3)	0.2233 (3)	0.4030 (2)	0.0582 (8)	
C7	0.2823 (4)	0.4809 (4)	0.2244 (3)	0.0739 (10)	
C8	0.4239 (5)	0.1142 (4)	0.4044 (3)	0.0839 (11)	
O1	0.0710 (4)	0.2287 (3)	0.7892 (2)	0.1067 (10)	
O2	0.2211 (3)	0.1013 (2)	0.6675 (2)	0.0774 (8)	
C9	0.1482 (4)	0.1403 (3)	0.7730 (3)	0.0673 (9)	
C10	0.1635 (4)	0.0674 (3)	0.8719 (3)	0.0765 (10)	
C11	0.2168 (3)	−0.0479 (3)	0.8489 (3)	0.0625 (8)	
C12	0.2269 (4)	−0.1235 (3)	0.9435 (3)	0.0713 (9)	
C13	0.2681 (4)	−0.2440 (4)	0.9157 (3)	0.0788 (11)	
C14	0.2767 (5)	−0.3265 (4)	1.0101 (4)	0.0994 (14)	
H2A	−0.017 (4)	0.428 (3)	0.589 (3)	0.072 (7)*	
H2B	0.036 (4)	0.321 (4)	0.640 (4)	0.095 (10)*	
H5	0.42270	0.27890	0.24760	0.0780*	
H7A	0.17580	0.41340	0.13310	0.1110*	
H7B	0.40180	0.50070	0.20330	0.1110*	
H7C	0.27990	0.58280	0.27620	0.1110*	
H8A	0.49650	0.14980	0.50460	0.1250*	
H8B	0.50880	0.11920	0.34600	0.1250*	
H8C	0.32460	0.00410	0.36340	0.1250*	
H2	0.212 (5)	0.160 (4)	0.610 (4)	0.125 (12)*	
H10	0.13270	0.10620	0.95800	0.0920*	
H11	0.25130	−0.08420	0.76420	0.0750*	
H12	0.20160	−0.08200	1.03180	0.0860*	
H13	0.29520	−0.28350	0.82780	0.0940*	
H14A	0.20900	−0.30130	1.07910	0.1480*	
H14B	0.40940	−0.28880	1.06350	0.1480*	
H14C	0.21730	−0.44230	0.94810	0.1480*	

### Atomic displacement parameters (Å^2^)

**Table:**

	*U*^11^	*U*^22^	*U*^33^	*U*^12^	*U*^13^	*U*^23^
N1	0.0710 (12)	0.0621 (12)	0.0571 (10)	0.0416 (10)	0.0276 (9)	0.0408 (9)
N2	0.129 (2)	0.1113 (19)	0.0869 (15)	0.0945 (18)	0.0700 (15)	0.0785 (15)
N3	0.0718 (12)	0.0614 (12)	0.0544 (10)	0.0374 (10)	0.0231 (9)	0.0399 (9)
C2	0.0704 (14)	0.0616 (13)	0.0540 (11)	0.0398 (12)	0.0244 (10)	0.0395 (10)
C4	0.0640 (13)	0.0602 (14)	0.0558 (12)	0.0265 (12)	0.0198 (10)	0.0382 (11)
C5	0.0774 (16)	0.0819 (17)	0.0676 (14)	0.0467 (14)	0.0384 (12)	0.0496 (13)
C6	0.0665 (14)	0.0676 (15)	0.0602 (13)	0.0393 (12)	0.0246 (11)	0.0387 (11)
C7	0.0909 (18)	0.0837 (18)	0.0752 (16)	0.0404 (15)	0.0363 (14)	0.0601 (15)
C8	0.109 (2)	0.106 (2)	0.0947 (19)	0.0792 (19)	0.0544 (17)	0.0657 (18)
O1	0.191 (2)	0.1244 (18)	0.1037 (15)	0.1172 (19)	0.0929 (16)	0.0885 (14)
O2	0.1068 (14)	0.1015 (14)	0.0864 (12)	0.0691 (12)	0.0541 (11)	0.0747 (11)
C9	0.0954 (18)	0.0668 (15)	0.0625 (14)	0.0436 (15)	0.0318 (13)	0.0433 (12)
C10	0.119 (2)	0.0733 (17)	0.0605 (14)	0.0492 (17)	0.0374 (14)	0.0455 (13)
C11	0.0651 (14)	0.0676 (15)	0.0638 (13)	0.0235 (12)	0.0200 (11)	0.0460 (12)
C12	0.0867 (18)	0.0665 (16)	0.0652 (14)	0.0271 (14)	0.0169 (12)	0.0453 (13)
C13	0.0751 (17)	0.092 (2)	0.102 (2)	0.0383 (16)	0.0352 (15)	0.0739 (18)
C14	0.099 (2)	0.093 (2)	0.128 (3)	0.0343 (18)	0.0218 (19)	0.087 (2)

### Geometric parameters (Å, º)

**Table:**

O1—C9	1.214 (5)	C7—H7A	0.9600
O2—C9	1.296 (4)	C8—H8C	0.9600
O2—H2	0.99 (4)	C8—H8A	0.9600
N1—C2	1.355 (4)	C8—H8B	0.9600
N1—C6	1.331 (3)	C9—C10	1.468 (4)
N2—C2	1.323 (4)	C10—C11	1.311 (4)
N3—C4	1.330 (3)	C11—C12	1.445 (4)
N3—C2	1.349 (3)	C12—C13	1.293 (5)
N2—H2A	0.89 (3)	C13—C14	1.496 (5)
N2—H2B	0.86 (4)	C10—H10	0.9300
C4—C7	1.500 (4)	C11—H11	0.9300
C4—C5	1.378 (4)	C12—H12	0.9300
C5—C6	1.376 (4)	C13—H13	0.9300
C6—C8	1.504 (5)	C14—H14A	0.9600
C5—H5	0.9300	C14—H14B	0.9600
C7—H7C	0.9600	C14—H14C	0.9600
C7—H7B	0.9600		
			
O1···N2	2.946 (4)	H2···C8	2.88 (4)
O2···N1	2.674 (3)	H2···C6	2.64 (4)
O2···C8	3.351 (4)	H2A···N3^iv^	2.19 (3)
O1···H14A^i^	2.9100	H2A···C4^iv^	3.09 (3)
O1···H2B	2.10 (4)	H2B···C9	2.92 (4)
O1···H14C^ii^	2.7200	H2B···O1	2.10 (4)
O1···H8C^iii^	2.8400	H2B···H2	2.43 (6)
O2···H11	2.4600	H5···H7B	2.4700
N1···O2	2.674 (3)	H5···H8B	2.4100
N2···N3^iv^	3.076 (4)	H5···H13^viii^	2.4400
N2···O1	2.946 (4)	H7B···H5	2.4700
N3···N2^iv^	3.076 (4)	H7C···H8A^ix^	2.4900
N1···H2	1.70 (4)	H8A···H7C^ix^	2.4900
N2···H2	2.89 (4)	H8B···H5	2.4100
N3···H2A^iv^	2.19 (3)	H8B···H11^viii^	2.4000
C2···C11^iii^	3.521 (3)	H8C···O1^iii^	2.8400
C7···C14^v^	3.425 (5)	H10···H12	2.4600
C8···O2	3.351 (4)	H10···H12^i^	2.5700
C11···C2^iii^	3.521 (3)	H11···O2	2.4600
C14···C7^vi^	3.425 (5)	H11···H13	2.4200
C2···H2	2.68 (4)	H11···H8B^viii^	2.4000
C4···H2A^iv^	3.09 (3)	H12···H10	2.4600
C6···H2	2.64 (4)	H12···H14A	2.4200
C8···H2	2.88 (4)	H12···H10^i^	2.5700
C9···H2B	2.92 (4)	H13···H11	2.4200
C10···H14B^vii^	3.0700	H13···H5^viii^	2.4400
H2···N2	2.89 (4)	H14A···H12	2.4200
H2···C2	2.68 (4)	H14A···O1^i^	2.9100
H2···N1	1.70 (4)	H14B···C10^vii^	3.0700
H2···H2B	2.43 (6)	H14C···O1^x^	2.7200
			
C9—O2—H2	110 (2)	C6—C8—H8A	109.00
C2—N1—C6	117.4 (2)	H8A—C8—H8C	110.00
C2—N3—C4	116.8 (2)	H8B—C8—H8C	109.00
C2—N2—H2B	118 (2)	H8A—C8—H8B	109.00
H2A—N2—H2B	119 (3)	O1—C9—C10	121.9 (3)
C2—N2—H2A	123.3 (19)	O2—C9—C10	114.8 (3)
N1—C2—N2	117.8 (2)	O1—C9—O2	123.3 (3)
N2—C2—N3	117.5 (3)	C9—C10—C11	125.5 (3)
N1—C2—N3	124.7 (2)	C10—C11—C12	125.8 (3)
C5—C4—C7	121.6 (2)	C11—C12—C13	125.5 (3)
N3—C4—C7	116.8 (2)	C12—C13—C14	126.8 (3)
N3—C4—C5	121.7 (2)	C9—C10—H10	117.00
C4—C5—C6	118.6 (3)	C11—C10—H10	117.00
N1—C6—C8	116.8 (2)	C10—C11—H11	117.00
C5—C6—C8	122.3 (3)	C12—C11—H11	117.00
N1—C6—C5	120.9 (3)	C11—C12—H12	117.00
C6—C5—H5	121.00	C13—C12—H12	117.00
C4—C5—H5	121.00	C12—C13—H13	117.00
C4—C7—H7A	109.00	C14—C13—H13	117.00
C4—C7—H7C	109.00	C13—C14—H14A	109.00
H7A—C7—H7B	109.00	C13—C14—H14B	109.00
C4—C7—H7B	109.00	C13—C14—H14C	109.00
H7B—C7—H7C	109.00	H14A—C14—H14B	110.00
H7A—C7—H7C	110.00	H14A—C14—H14C	110.00
C6—C8—H8B	109.00	H14B—C14—H14C	109.00
C6—C8—H8C	109.00		
			
C6—N1—C2—N2	178.8 (2)	C7—C4—C5—C6	179.5 (3)
C6—N1—C2—N3	−1.1 (3)	C4—C5—C6—N1	0.9 (4)
C2—N1—C6—C5	0.1 (3)	C4—C5—C6—C8	−179.3 (3)
C2—N1—C6—C8	−179.7 (2)	O1—C9—C10—C11	168.8 (3)
C4—N3—C2—N1	1.1 (3)	O2—C9—C10—C11	−10.4 (5)
C4—N3—C2—N2	−178.9 (2)	C9—C10—C11—C12	−178.1 (3)
C2—N3—C4—C5	0.0 (3)	C10—C11—C12—C13	175.5 (3)
C2—N3—C4—C7	179.6 (2)	C11—C12—C13—C14	−179.0 (3)
N3—C4—C5—C6	−1.0 (4)		

Symmetry codes: (i) −*x*, −*y*, −*z*+2; (ii) *x*, *y*+1, *z*; (iii) −*x*, −*y*, −*z*+1; (iv) −*x*, −*y*+1, −*z*+1; (v) *x*, *y*+1, *z*−1; (vi) *x*, *y*−1, *z*+1; (vii) −*x*+1, −*y*, −*z*+2; (viii) −*x*+1, −*y*, −*z*+1; (ix) −*x*+1, −*y*+1, −*z*+1; (x) *x*, *y*−1, *z*.

### Hydrogen-bond geometry (Å, º)

**Table:**

*D*—H···*A*	*D*—H	H···*A*	*D*···*A*	*D*—H···*A*
O2—H2···N1	0.99 (4)	1.70 (4)	2.674 (3)	167 (4)
N2—H2*A*···N3^iv^	0.89 (3)	2.19 (3)	3.076 (4)	176 (2)
N2—H2*B*···O1	0.86 (4)	2.10 (4)	2.946 (4)	171 (3)

Symmetry code: (iv) −*x*, −*y*+1, −*z*+1.

## References

[bb1] Bernstein, J., Davis, R. E., Shimoni, L. & Chang, N.-L. (1995). *Angew. Chem. Int. Ed. Engl.* **34**, 1555–1573.

[bb2] Bruker (2008). *APEX2*, *SAINT* and *SADABS* Bruker AXS Inc., Madison, Wisconsin, USA.

[bb3] Cason, C. J. (2004). *POV-RAY* Persistence of Vision Raytracer Pty. Ltd, Victoria, Australia.

[bb4] Ebenezer, S. & Muthiah, P. T. (2010). *Acta Cryst.* E**66**, o2634–o2635. 10.1107/S1600536810037724 PMC2983259 21587607

[bb5] Ebenezer, S. & Muthiah, P. T. (2012). *Cryst. Growth Des.* **12**, 3766–3785.

[bb6] Etter, M. C. (1990). *Acc. Chem. Res.* **23**, 120–126.

[bb7] Hunt, W. E., Schwalbe, C. H., Bird, K. & Mallinson, P. D. (1980). *J. Biochem.* **187**, 533–536. 10.1042/bj1870533 PMC1161822 6893149

[bb8] Macrae, C. F., Bruno, I. J., Chisholm, J. A., Edgington, P. R., McCabe, P., Pidcock, E., Rodriguez-Monge, L., Taylor, R., van de Streek, J. & Wood, P. A. (2008). *J. Appl. Cryst.* **41**, 466–470.

[bb9] Sheldrick, G. M. (2008). *Acta Cryst.* A**64**, 112–122. 10.1107/S0108767307043930 18156677

[bb10] Spek, A. L. (2009). *Acta Cryst.* D**65**, 148–155. 10.1107/S090744490804362X PMC2631630 19171970

[bb11] Thanigaimani, K., Muthiah, P. T. & Lynch, D. E. (2007). *Acta Cryst.* E**63**, o4450–o4451. 10.1107/S0108270107005677 17478916

